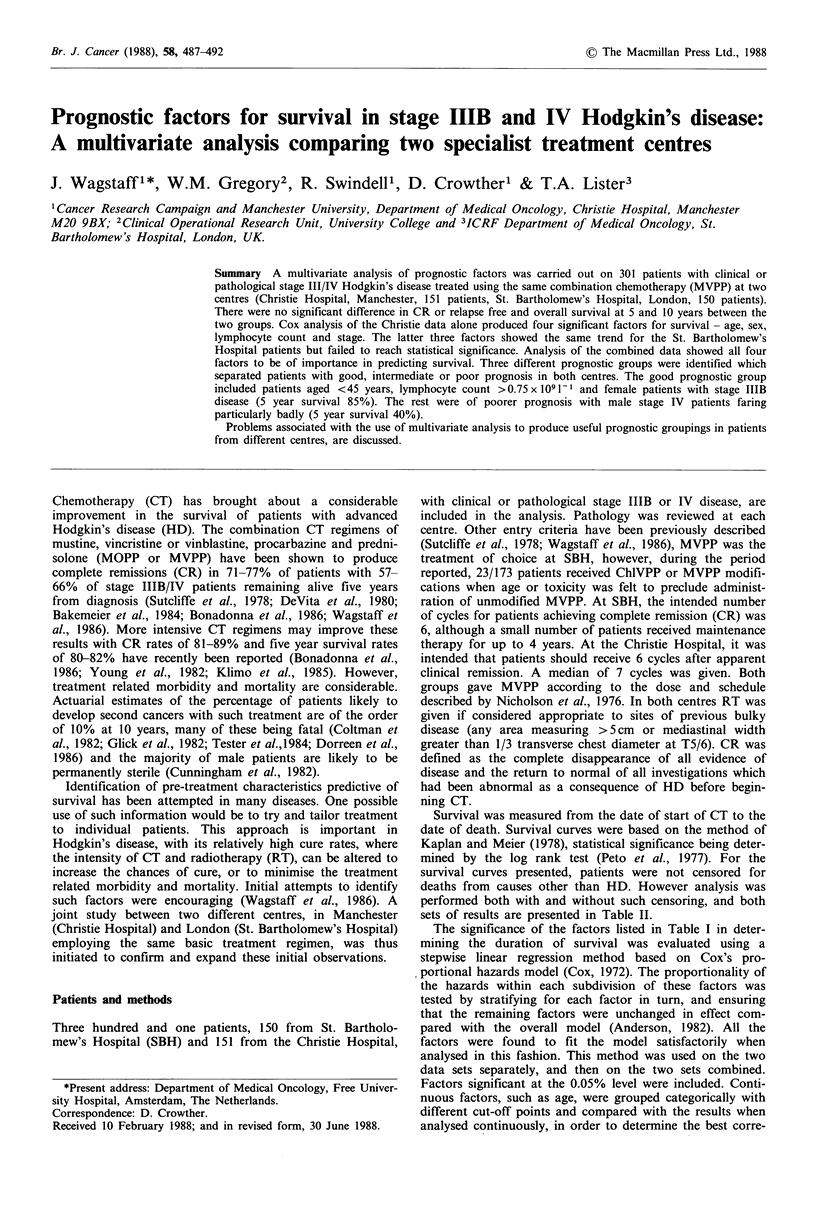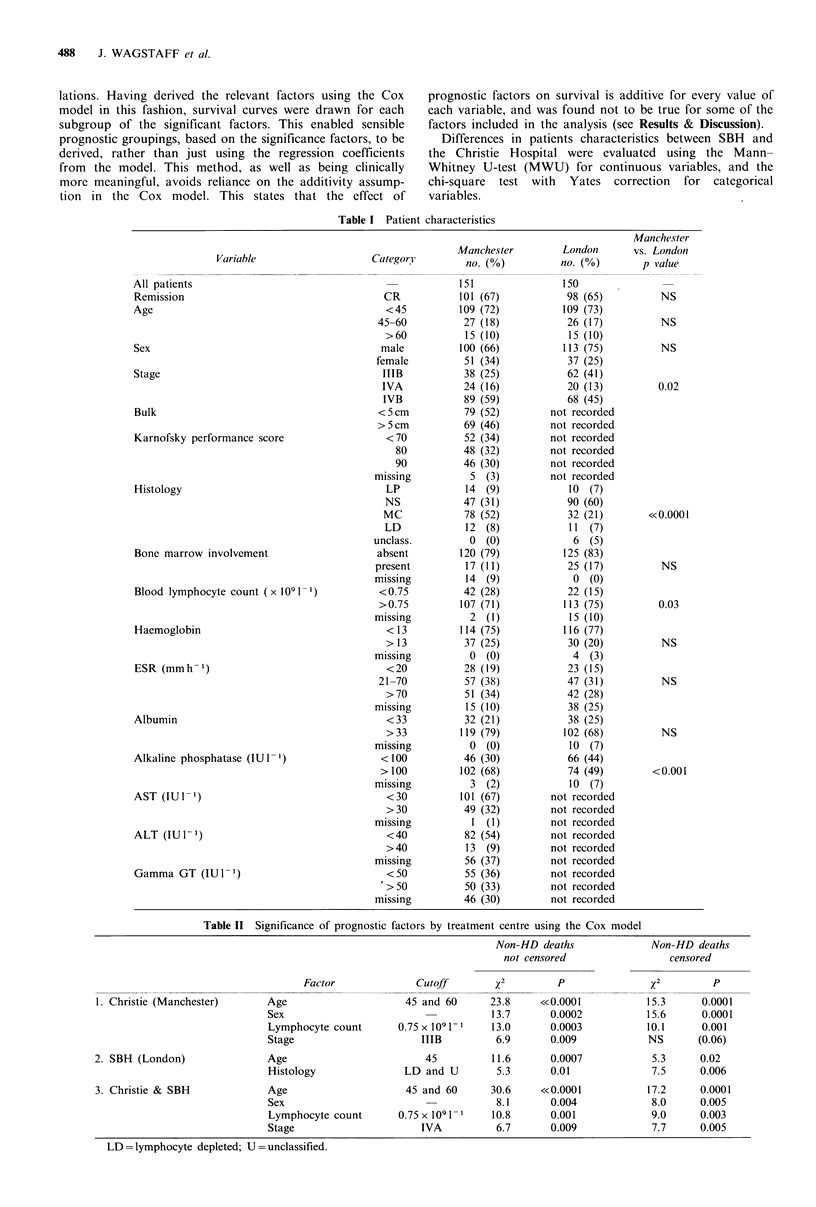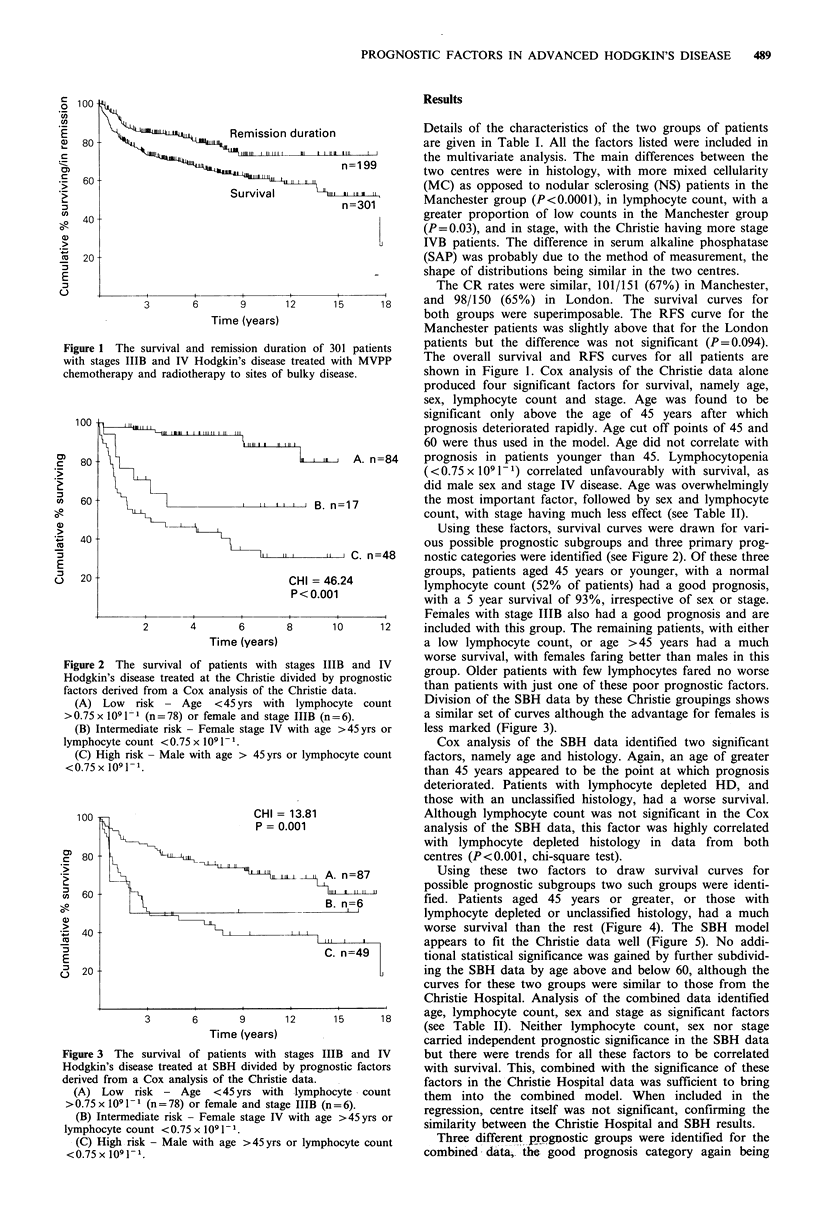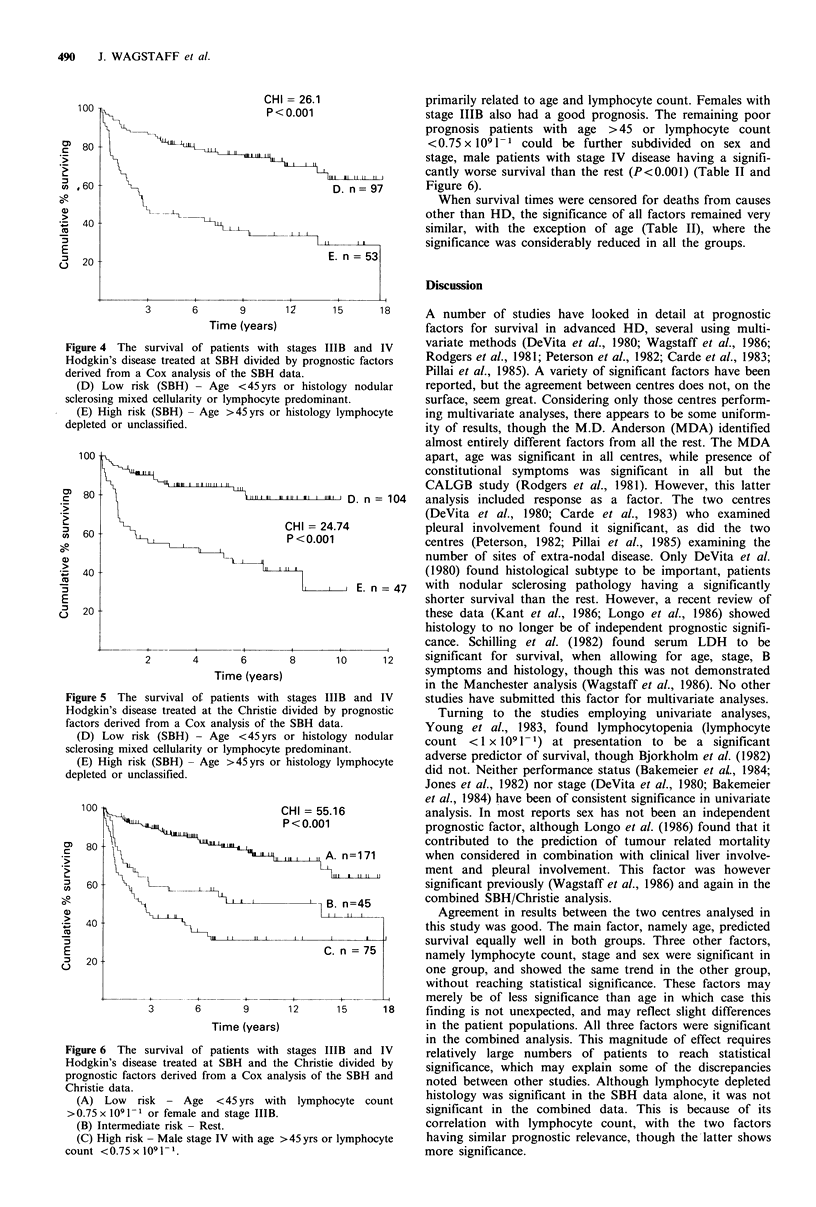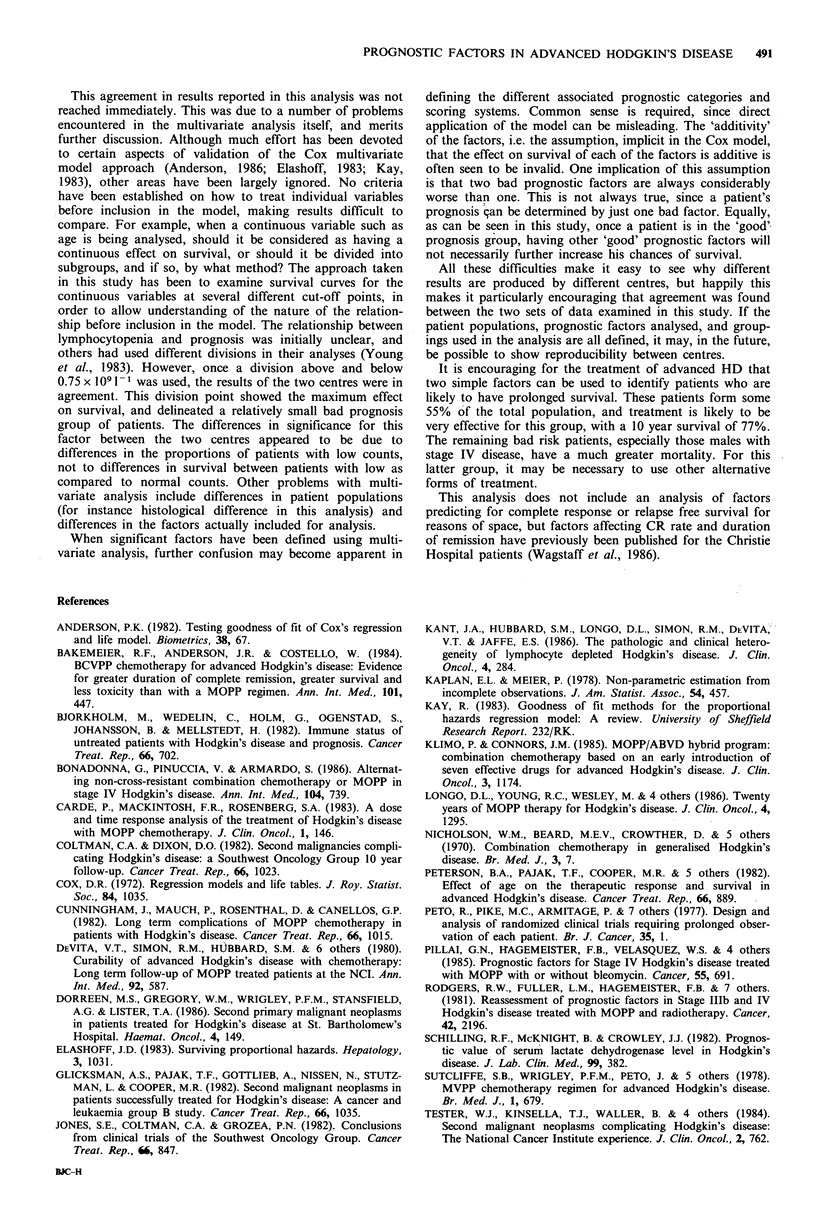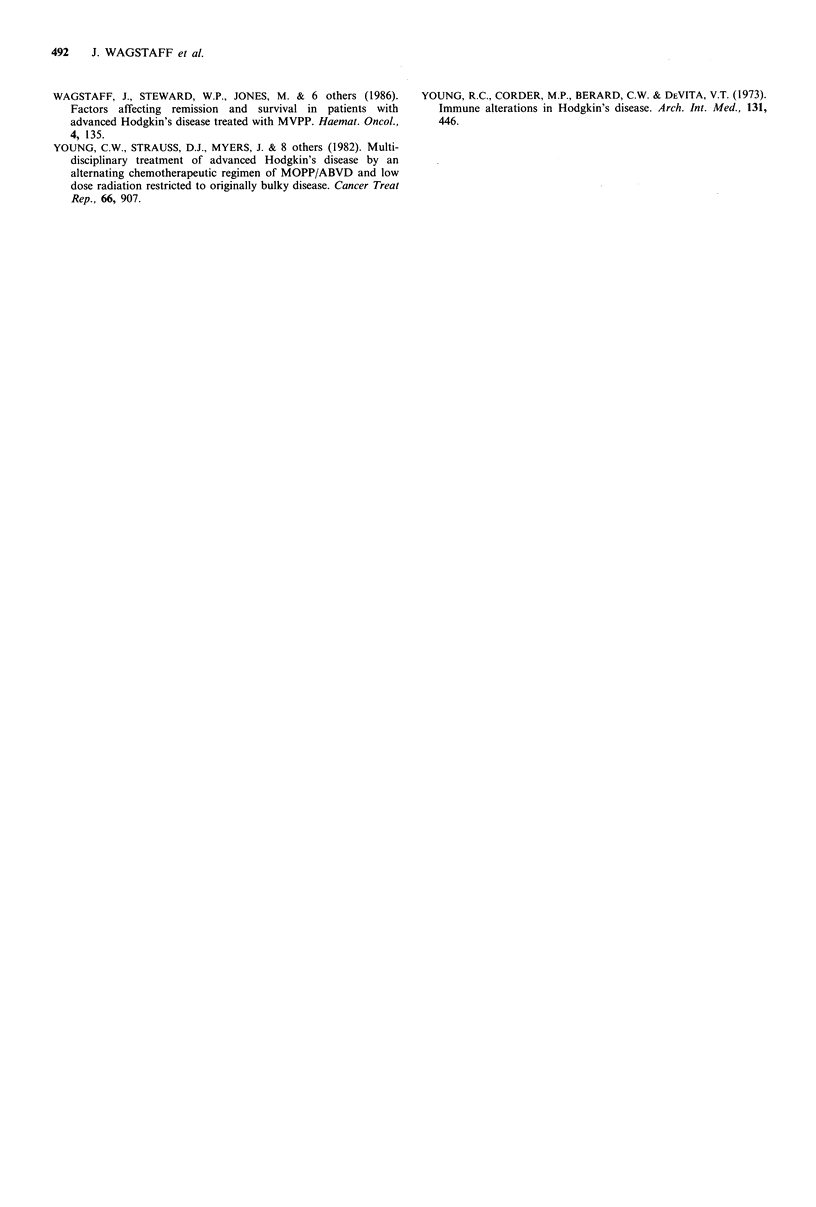# Prognostic factors for survival in stage IIIB and IV Hodgkin's disease: a multivariate analysis comparing two specialist treatment centres.

**DOI:** 10.1038/bjc.1988.246

**Published:** 1988-10

**Authors:** J. Wagstaff, W. M. Gregory, R. Swindell, D. Crowther, T. A. Lister

**Affiliations:** Cancer Research Campaign, Christie Hospital, Manchester, UK.

## Abstract

A multivariate analysis of prognostic factors was carried out on 301 patients with clinical or pathological stage III/IV Hodgkin's disease treated using the same combination chemotherapy (MVPP) at two centres (Christie Hospital, Manchester, 151 patients, St. Bartholomew's Hospital, London, 150 patients). There were no significant difference in CR or relapse free and overall survival at 5 and 10 years between the two groups. Cox analysis of the Christie data alone produced four significant factors for survival - age, sex, lymphocyte count and stage. The latter three factors showed the same trend for the St. Bartholomew's Hospital patients but failed to reach statistical significance. Analysis of the combined data showed all four factors to be of importance in predicting survival. Three different prognostic groups were identified which separated patients with good, intermediate or poor prognosis in both centres. The good prognostic group included patients aged less than 45 years, lymphocyte count greater than 0.75 x 10(9) l-1 and female patients with stage IIIB disease (5 year survival 85%). The rest were of poorer prognosis with male stage IV patients faring particularly badly (5 year survival 40%). Problems associated with the use of multivariate analysis to produce useful prognostic groupings in patients from different centres, are discussed.


*Br. J. Cancer* (1988), **58**, 487–492 © The Macmillan Press Ltd., 1988

### Prognostic factors for survival in stage IIIB and IV Hodgkin's disease: A multivariate analysis comparing two specialist treatment centres

J. Wagstaff[1]*, W.M. Gregory[2], R. Swindell[1], D. Crowther[1] & T.A. Lister[3]

[1]*Cancer Research Campaign and Manchester University, Department of Medical Oncology, Christie Hospital, Manchester M20 9BX;* [2]*Clinical Operational Research Unit, University College and* [3]*ICRF Department of Medical Oncology, St. Bartholomew's Hospital, London, UK.*

**Summary** A multivariate analysis of prognostic factors was carried out on 301 patients with clinical or pathological stage III/IV Hodgkin's disease treated using the same combination chemotherapy (MVPP) at two centres (Christie Hospital, Manchester, 151 patients, St. Bartholomew's Hospital, London, 150 patients). There were no significant difference in CR or relapse free and overall survival at 5 and 10 years between the two groups. Cox analysis of the Christie data alone produced four significant factors for survival – age, sex, lymphocyte count and stage. The latter three factors showed the same trend for the St. Bartholomew's Hospital patients but failed to reach statistical significance. Analysis of the combined data showed all four factors to be of importance in predicting survival. Three different prognostic groups were identified which separated patients with good, intermediate or poor prognosis in both centres. The good prognostic group included patients aged <45 years, lymphocyte count $>0.75\times10^9 l^{-1}$ and female patients with stage IIIB disease (5 year survival 85%). The rest were of poorer prognosis with male stage IV patients faring particularly badly (5 year survival 40%).

Problems associated with the use of multivariate analysis to produce useful prognostic groupings in patients from different centres, are discussed.

Chemotherapy (CT) has brought about a considerable improvement in the survival of patients with advanced Hodgkin's disease (HD). The combination CT regimens of mustine, vincristine or vinblastine, procarbazine and prednisolone (MOPP or MVPP) have been shown to produce complete remissions (CR) in 71–77% of patients with 57–66% of stage IIIB/IV patients remaining alive five years from diagnosis (Sutcliffe *et al.*, 1978; DeVita *et al.*, 1980; Bakemeier *et al.*, 1984; Bonadonna *et al.*, 1986; Wagstaff *et al.*, 1986). More intensive CT regimens may improve these results with CR rates of 81–89% and five year survival rates of 80–82% have recently been reported (Bonadonna *et al.*, 1986; Young *et al.*, 1982; Klimo *et al.*, 1985). However, treatment related morbidity and mortality are considerable. Actuarial estimates of the percentage of patients likely to develop second cancers with such treatment are of the order of 10% at 10 years, many of these being fatal (Coltman *et al.*, 1982; Glick *et al.*, 1982; Tester *et al.*,1984; Dorreen *et al.*, 1986) and the majority of male patients are likely to be permanently sterile (Cunningham *et al.*, 1982).

Identification of pre-treatment characteristics predictive of survival has been attempted in many diseases. One possible use of such information would be to try and tailor treatment to individual patients. This approach is important in Hodgkin's disease, with its relatively high cure rates, where the intensity of CT and radiotherapy (RT), can be altered to increase the chances of cure, or to minimise the treatment related morbidity and mortality. Initial attempts to identify such factors were encouraging (Wagstaff *et al.*, 1986). A joint study between two different centres, in Manchester (Christie Hospital) and London (St. Bartholomew's Hospital) employing the same basic treatment regimen, was thus initiated to confirm and expand these initial observations.

### Patients and methods

Three hundred and one patients, 150 from St. Bartholomew's Hospital (SBH) and 151 from the Christie Hospital, with clinical or pathological stage IIIB or IV disease, are included in the analysis. Pathology was reviewed at each centre. Other entry criteria have been previously described (Sutcliffe *et al.*, 1978; Wagstaff *et al.*, 1986), MVPP was the treatment of choice at SBH, however, during the period reported, 23/173 patients received ChlVPP or MVPP modifications when age or toxicity was felt to preclude administration of unmodified MVPP. At SBH, the intended number of cycles for patients achieving complete remission (CR) was 6, although a small number of patients received maintenance therapy for up to 4 years. At the Christie Hospital, it was intended that patients should receive 6 cycles after apparent clinical remission. A median of 7 cycles was given. Both groups gave MVPP according to the dose and schedule described by Nicholson *et al.*, 1976. In both centres RT was given if considered appropriate to sites of previous bulky disease (any area measuring >5cm or mediastinal width greater than 1/3 transverse chest diameter at T5/6). CR was defined as the complete disappearance of all evidence of disease and the return to normal of all investigations which had been abnormal as a consequence of HD before beginning CT.

Survival was measured from the date of start of CT to the date of death. Survival curves were based on the method of Kaplan and Meier (1978), statistical significance being determined by the log rank test (Peto *et al.*, 1977). For the survival curves presented, patients were not censored for deaths from causes other than HD. However analysis was performed both with and without such censoring, and both sets of results are presented in Table II.

The significance of the factors listed in Table I in determining the duration of survival was evaluated using a stepwise linear regression method based on Cox's proportional hazards model (Cox, 1972). The proportionality of the hazards within each subdivision of these factors was tested by stratifying for each factor in turn, and ensuring that the remaining factors were unchanged in effect compared with the overall model (Anderson, 1982). All the factors were found to fit the model satisfactorily when analysed in this fashion. This method was used on the two data sets separately, and then on the two sets combined. Factors significant at the 0.05% level were included. Continuous factors, such as age, were grouped categorically with different cut-off points and compared with the results when analysed continuously, in order to determine the best corre-

*Present address: Department of Medical Oncology, Free University Hospital, Amsterdam, The Netherlands.
Correspondence: D. Crowther.
Received 10 February 1988; and in revised form, 30 June 1988.

lations. Having derived the relevant factors using the Cox model in this fashion, survival curves were drawn for each subgroup of the significant factors. This enabled sensible prognostic groupings, based on the significance factors, to be derived, rather than just using the regression coefficients from the model. This method, as well as being clinically more meaningful, avoids reliance on the additivity assumption in the Cox model. This states that the effect of prognostic factors on survival is additive for every value of each variable, and was found not to be true for some of the factors included in the analysis (see **Results & Discussion**).

Differences in patients characteristics between SBH and the Christie Hospital were evaluated using the Mann–Whitney U-test (MWU) for continuous variables, and the chi-square test with Yates correction for categorical variables.

**Table I** Patient characteristics

| *Variable* | *Category* | *Manchester no. (%)* | *London no. (%)* | *Manchester vs. London p value* |
|---|---|---|---|---|
| All patients | — | 151 | 150 | — |
| Remission | CR | 101 (67) | 98 (65) | NS |
| Age | <45 | 109 (72) | 109 (73) | |
| | 45–60 | 27 (18) | 26 (17) | NS |
| | >60 | 15 (10) | 15 (10) | |
| Sex | male | 100 (66) | 113 (75) | NS |
| | female | 51 (34) | 37 (25) | |
| Stage | IIIB | 38 (25) | 62 (41) | |
| | IVA | 24 (16) | 20 (13) | 0.02 |
| | IVB | 89 (59) | 68 (45) | |
| Bulk | <5 cm | 79 (52) | not recorded | |
| | >5 cm | 69 (46) | not recorded | |
| Karnofsky performance score | <70 | 52 (34) | not recorded | |
| | 80 | 48 (32) | not recorded | |
| | 90 | 46 (30) | not recorded | |
| | missing | 5 (3) | not recorded | |
| Histology | LP | 14 (9) | 10 (7) | |
| | NS | 47 (31) | 90 (60) | |
| | MC | 78 (52) | 32 (21) | ≪0.0001 |
| | LD | 12 (8) | 11 (7) | |
| | unclass. | 0 (0) | 6 (5) | |
| Bone marrow involvement | absent | 120 (79) | 125 (83) | |
| | present | 17 (11) | 25 (17) | NS |
| | missing | 14 (9) | 0 (0) | |
| Blood lymphocyte count ($\times 10^9 l^{-1}$) | <0.75 | 42 (28) | 22 (15) | |
| | >0.75 | 107 (71) | 113 (75) | 0.03 |
| | missing | 2 (1) | 15 (10) | |
| Haemoglobin | <13 | 114 (75) | 116 (77) | |
| | >13 | 37 (25) | 30 (20) | NS |
| | missing | 0 (0) | 4 (3) | |
| ESR ($mm\,h^{-1}$) | <20 | 28 (19) | 23 (15) | |
| | 21–70 | 57 (38) | 47 (31) | NS |
| | >70 | 51 (34) | 42 (28) | |
| | missing | 15 (10) | 38 (25) | |
| Albumin | <33 | 32 (21) | 38 (25) | |
| | >33 | 119 (79) | 102 (68) | NS |
| | missing | 0 (0) | 10 (7) | |
| Alkaline phosphatase ($IU\,l^{-1}$) | <100 | 46 (30) | 66 (44) | |
| | >100 | 102 (68) | 74 (49) | <0.001 |
| | missing | 3 (2) | 10 (7) | |
| AST ($IU\,l^{-1}$) | <30 | 101 (67) | not recorded | |
| | >30 | 49 (32) | not recorded | |
| | missing | 1 (1) | not recorded | |
| ALT ($IU\,l^{-1}$) | <40 | 82 (54) | not recorded | |
| | >40 | 13 (9) | not recorded | |
| | missing | 56 (37) | not recorded | |
| Gamma GT ($IU\,l^{-1}$) | <50 | 55 (36) | not recorded | |
| | >50 | 50 (33) | not recorded | |
| | missing | 46 (30) | not recorded | |

**Table II** Significance of prognostic factors by treatment centre using the Cox model

| | | | *Non-HD deaths not censored* | | *Non-HD deaths censored* | |
|---|---|---|---|---|---|---|
| | *Factor* | *Cutoff* | $\chi^2$ | *P* | $\chi^2$ | *P* |
| 1. Christie (Manchester) | Age | 45 and 60 | 23.8 | ≪0.0001 | 15.3 | 0.0001 |
| | Sex | — | 13.7 | 0.0002 | 15.6 | 0.0001 |
| | Lymphocyte count | $0.75 \times 10^9 l^{-1}$ | 13.0 | 0.0003 | 10.1 | 0.001 |
| | Stage | IIIB | 6.9 | 0.009 | NS | (0.06) |
| 2. SBH (London) | Age | 45 | 11.6 | 0.0007 | 5.3 | 0.02 |
| | Histology | LD and U | 5.3 | 0.01 | 7.5 | 0.006 |
| 3. Christie & SBH | Age | 45 and 60 | 30.6 | ≪0.0001 | 17.2 | 0.0001 |
| | Sex | — | 8.1 | 0.004 | 8.0 | 0.005 |
| | Lymphocyte count | $0.75 \times 10^9 l^{-1}$ | 10.8 | 0.001 | 9.0 | 0.003 |
| | Stage | IVA | 6.7 | 0.009 | 7.7 | 0.005 |

LD = lymphocyte depleted; U = unclassified.

**Figure 1** The survival and remission duration of 301 patients with stages IIIB and IV Hodgkin's disease treated with MVPP chemotherapy and radiotherapy to sites of bulky disease.

**Figure 2** The survival of patients with stages IIIB and IV Hodgkin's disease treated at the Christie divided by prognostic factors derived from a Cox analysis of the Christie data.

(A) Low risk – Age <45 yrs with lymphocyte count $>0.75\times10^{9}l^{-1}$ (n=78) or female and stage IIIB (n=6).

(B) Intermediate risk – Female stage IV with age >45 yrs or lymphocyte count $<0.75\times10^{9}l^{-1}$.

(C) High risk – Male with age > 45 yrs or lymphocyte count $<0.75\times10^{9}l^{-1}$.

**Figure 3** The survival of patients with stages IIIB and IV Hodgkin's disease treated at SBH divided by prognostic factors derived from a Cox analysis of the Christie data.

(A) Low risk – Age <45 yrs with lymphocyte count $>0.75\times10^{9}l^{-1}$ (n=78) or female and stage IIIB (n=6).

(B) Intermediate risk – Female stage IV with age >45 yrs or lymphocyte count $<0.75\times10^{9}l^{-1}$.

(C) High risk – Male with age >45 yrs or lymphocyte count $<0.75\times10^{9}l^{-1}$.

### Results

Details of the characteristics of the two groups of patients are given in Table I. All the factors listed were included in the multivariate analysis. The main differences between the two centres were in histology, with more mixed cellularity (MC) as opposed to nodular sclerosing (NS) patients in the Manchester group ($P<0.0001$), in lymphocyte count, with a greater proportion of low counts in the Manchester group ($P=0.03$), and in stage, with the Christie having more stage IVB patients. The difference in serum alkaline phosphatase (SAP) was probably due to the method of measurement, the shape of distributions being similar in the two centres.

The CR rates were similar, 101/151 (67%) in Manchester, and 98/150 (65%) in London. The survival curves for both groups were superimposable. The RFS curve for the Manchester patients was slightly above that for the London patients but the difference was not significant ($P=0.094$). The overall survival and RFS curves for all patients are shown in Figure 1. Cox analysis of the Christie data alone produced four significant factors for survival, namely age, sex, lymphocyte count and stage. Age was found to be significant only above the age of 45 years after which prognosis deteriorated rapidly. Age cut off points of 45 and 60 were thus used in the model. Age did not correlate with prognosis in patients younger than 45. Lymphocytopenia ($<0.75\times10^{9}l^{-1}$) correlated unfavourably with survival, as did male sex and stage IV disease. Age was overwhelmingly the most important factor, followed by sex and lymphocyte count, with stage having much less effect (see Table II).

Using these factors, survival curves were drawn for various possible prognostic subgroups and three primary prognostic categories were identified (see Figure 2). Of these three groups, patients aged 45 years or younger, with a normal lymphocyte count (52% of patients) had a good prognosis, with a 5 year survival of 93%, irrespective of sex or stage. Females with stage IIIB also had a good prognosis and are included with this group. The remaining patients, with either a low lymphocyte count, or age >45 years had a much worse survival, with females faring better than males in this group. Older patients with few lymphocytes fared no worse than patients with just one of these poor prognostic factors. Division of the SBH data by these Christie groupings shows a similar set of curves although the advantage for females is less marked (Figure 3).

Cox analysis of the SBH data identified two significant factors, namely age and histology. Again, an age of greater than 45 years appeared to be the point at which prognosis deteriorated. Patients with lymphocyte depleted HD, and those with an unclassified histology, had a worse survival. Although lymphocyte count was not significant in the Cox analysis of the SBH data, this factor was highly correlated with lymphocyte depleted histology in data from both centres ($P<0.001$, chi-square test).

Using these two factors to draw survival curves for possible prognostic subgroups two such groups were identified. Patients aged 45 years or greater, or those with lymphocyte depleted or unclassified histology, had a much worse survival than the rest (Figure 4). The SBH model appears to fit the Christie data well (Figure 5). No additional statistical significance was gained by further subdividing the SBH data by age above and below 60, although the curves for these two groups were similar to those from the Christie Hospital. Analysis of the combined data identified age, lymphocyte count, sex and stage as significant factors (see Table II). Neither lymphocyte count, sex nor stage carried independent prognostic significance in the SBH data but there were trends for all these factors to be correlated with survival. This, combined with the significance of these factors in the Christie Hospital data was sufficient to bring them into the combined model. When included in the regression, centre itself was not significant, confirming the similarity between the Christie Hospital and SBH results.

Three different prognostic groups were identified for the combined data, the good prognosis category again being

**Figure 4** The survival of patients with stages IIIB and IV Hodgkin's disease treated at SBH divided by prognostic factors derived from a Cox analysis of the SBH data.

(D) Low risk (SBH) – Age <45 yrs or histology nodular sclerosing mixed cellularity or lymphocyte predominant.

(E) High risk (SBH) – Age >45 yrs or histology lymphocyte depleted or unclassified.

**Figure 5** The survival of patients with stages IIIB and IV Hodgkin's disease treated at the Christie divided by prognostic factors derived from a Cox analysis of the SBH data.

(D) Low risk (SBH) – Age <45 yrs or histology nodular sclerosing mixed cellularity or lymphocyte predominant.

(E) High risk (SBH) – Age >45 yrs or histology lymphocyte depleted or unclassified.

**Figure 6** The survival of patients with stages IIIB and IV Hodgkin's disease treated at SBH and the Christie divided by prognostic factors derived from a Cox analysis of the SBH and Christie data.

(A) Low risk – Age <45 yrs with lymphocyte count $>0.75\times10^9 l^{-1}$ or female and stage IIIB.

(B) Intermediate risk – Rest.

(C) High risk – Male stage IV with age >45 yrs or lymphocyte count $<0.75\times10^9 l^{-1}$.

primarily related to age and lymphocyte count. Females with stage IIIB also had a good prognosis. The remaining poor prognosis patients with age >45 or lymphocyte count $<0.75\times10^9 l^{-1}$ could be further subdivided on sex and stage, male patients with stage IV disease having a significantly worse survival than the rest ($P<0.001$) (Table II and Figure 6).

When survival times were censored for deaths from causes other than HD, the significance of all factors remained very similar, with the exception of age (Table II), where the significance was considerably reduced in all the groups.

### Discussion

A number of studies have looked in detail at prognostic factors for survival in advanced HD, several using multivariate methods (DeVita *et al.*, 1980; Wagstaff *et al.*, 1986; Rodgers *et al.*, 1981; Peterson *et al.*, 1982; Carde *et al.*, 1983; Pillai *et al.*, 1985). A variety of significant factors have been reported, but the agreement between centres does not, on the surface, seem great. Considering only those centres performing multivariate analyses, there appears to be some uniformity of results, though the M.D. Anderson (MDA) identified almost entirely different factors from all the rest. The MDA apart, age was significant in all centres, while presence of constitutional symptoms was significant in all but the CALGB study (Rodgers *et al.*, 1981). However, this latter analysis included response as a factor. The two centres (DeVita *et al.*, 1980; Carde *et al.*, 1983) who examined pleural involvement found it significant, as did the two centres (Peterson, 1982; Pillai *et al.*, 1985) examining the number of sites of extra-nodal disease. Only DeVita *et al.* (1980) found histological subtype to be important, patients with nodular sclerosing pathology having a significantly shorter survival than the rest. However, a recent review of these data (Kant *et al.*, 1986; Longo *et al.*, 1986) showed histology to no longer be of independent prognostic significance. Schilling *et al.* (1982) found serum LDH to be significant for survival, when allowing for age, stage, B symptoms and histology, though this was not demonstrated in the Manchester analysis (Wagstaff *et al.*, 1986). No other studies have submitted this factor for multivariate analyses.

Turning to the studies employing univariate analyses, Young *et al.*, 1983, found lymphocytopenia (lymphocyte count $<1\times10^9 l^{-1}$) at presentation to be a significant adverse predictor of survival, though Bjorkholm *et al.* (1982) did not. Neither performance status (Bakemeier *et al.*, 1984; Jones *et al.*, 1982) nor stage (DeVita *et al.*, 1980; Bakemeier *et al.*, 1984) have been of consistent significance in univariate analysis. In most reports sex has not been an independent prognostic factor, although Longo *et al.* (1986) found that it contributed to the prediction of tumour related mortality when considered in combination with clinical liver involvement and pleural involvement. This factor was however significant previously (Wagstaff *et al.*, 1986) and again in the combined SBH/Christie analysis.

Agreement in results between the two centres analysed in this study was good. The main factor, namely age, predicted survival equally well in both groups. Three other factors, namely lymphocyte count, stage and sex were significant in one group, and showed the same trend in the other group, without reaching statistical significance. These factors may merely be of less significance than age in which case this finding is not unexpected, and may reflect slight differences in the patient populations. All three factors were significant in the combined analysis. This magnitude of effect requires relatively large numbers of patients to reach statistical significance, which may explain some of the discrepancies noted between other studies. Although lymphocyte depleted histology was significant in the SBH data alone, it was not significant in the combined data. This is because of its correlation with lymphocyte count, with the two factors having similar prognostic relevance, though the latter shows more significance.

This agreement in results reported in this analysis was not reached immediately. This was due to a number of problems encountered in the multivariate analysis itself, and merits further discussion. Although much effort has been devoted to certain aspects of validation of the Cox multivariate model approach (Anderson, 1986; Elashoff, 1983; Kay, 1983), other areas have been largely ignored. No criteria have been established on how to treat individual variables before inclusion in the model, making results difficult to compare. For example, when a continuous variable such as age is being analysed, should it be considered as having a continuous effect on survival, or should it be divided into subgroups, and if so, by what method? The approach taken in this study has been to examine survival curves for the continuous variables at several different cut-off points, in order to allow understanding of the nature of the relationship before inclusion in the model. The relationship between lymphocytopenia and prognosis was initially unclear, and others had used different divisions in their analyses (Young *et al.*, 1983). However, once a division above and below $0.75 \times 10^9 l^{-1}$ was used, the results of the two centres were in agreement. This division point showed the maximum effect on survival, and delineated a relatively small bad prognosis group of patients. The differences in significance for this factor between the two centres appeared to be due to differences in the proportions of patients with low counts, not to differences in survival between patients with low as compared to normal counts. Other problems with multivariate analysis include differences in patient populations (for instance histological difference in this analysis) and differences in the factors actually included for analysis.

When significant factors have been defined using multivariate analysis, further confusion may become apparent in defining the different associated prognostic categories and scoring systems. Common sense is required, since direct application of the model can be misleading. The 'additivity' of the factors, i.e. the assumption, implicit in the Cox model, that the effect on survival of each of the factors is additive is often seen to be invalid. One implication of this assumption is that two bad prognostic factors are always considerably worse than one. This is not always true, since a patient's prognosis can be determined by just one bad factor. Equally, as can be seen in this study, once a patient is in the 'good' prognosis group, having other 'good' prognostic factors will not necessarily further increase his chances of survival.

All these difficulties make it easy to see why different results are produced by different centres, but happily this makes it particularly encouraging that agreement was found between the two sets of data examined in this study. If the patient populations, prognostic factors analysed, and groupings used in the analysis are all defined, it may, in the future, be possible to show reproducibility between centres.

It is encouraging for the treatment of advanced HD that two simple factors can be used to identify patients who are likely to have prolonged survival. These patients form some 55% of the total population, and treatment is likely to be very effective for this group, with a 10 year survival of 77%. The remaining bad risk patients, especially those males with stage IV disease, have a much greater mortality. For this latter group, it may be necessary to use other alternative forms of treatment.

This analysis does not include an analysis of factors predicting for complete response or relapse free survival for reasons of space, but factors affecting CR rate and duration of remission have previously been published for the Christie Hospital patients (Wagstaff *et al.*, 1986).

**References**

ANDERSON, P.K. (1982). Testing goodness of fit of Cox's regression and life model. *Biometrics*, **38**, 67.

BAKEMEIER, R.F., ANDERSON, J.R. & COSTELLO, W. (1984). BCVPP chemotherapy for advanced Hodgkin's disease: Evidence for greater duration of complete remission, greater survival and less toxicity than with a MOPP regimen. *Ann. Int. Med.*, **101**, 447.

BJORKHOLM, M., WEDELIN, C., HOLM, G., OGENSTAD, S., JOHANSSON, B. & MELLSTEDT, H. (1982). Immune status of untreated patients with Hodgkin's disease and prognosis. *Cancer Treat. Rep.*, **66**, 702.

BONADONNA, G., PINUCCIA, V. & ARMARDO, S. (1986). Alternating non-cross-resistant combination chemotherapy or MOPP in stage IV Hodgkin's disease. *Ann. Int. Med.*, **104**, 739.

CARDE, P., MACKINTOSH, F.R., ROSENBERG, S.A. (1983). A dose and time response analysis of the treatment of Hodgkin's disease with MOPP chemotherapy. *J. Clin. Oncol.*, **1**, 146.

COLTMAN, C.A. & DIXON, D.O. (1982). Second malignancies complicating Hodgkin's disease: a Southwest Oncology Group 10 year follow-up. *Cancer Treat. Rep.*, **66**, 1023.

COX, D.R. (1972). Regression models and life tables. *J. Roy. Statist. Soc.*, **84**, 1035.

CUNNINGHAM, J., MAUCH, P., ROSENTHAL, D. & CANELLOS, G.P. (1982). Long term complications of MOPP chemotherapy in patients with Hodgkin's disease. *Cancer Treat. Rep.*, **66**, 1015.

DEVITA, V.T., SIMON, R.M., HUBBARD, S.M. & 6 others (1980). Curability of advanced Hodgkin's disease with chemotherapy: Long term follow-up of MOPP treated patients at the NCI. *Ann. Int. Med.*, **92**, 587.

DORREEN, M.S., GREGORY, W.M., WRIGLEY, P.F.M., STANSFIELD, A.G. & LISTER, T.A. (1986). Second primary malignant neoplasms in patients treated for Hodgkin's disease at St. Bartholomew's Hospital. *Haemat. Oncol.*, **4**, 149.

ELASHOFF, J.D. (1983). Surviving proportional hazards. *Hepatology*, **3**, 1031.

GLICKSMAN, A.S., PAJAK, T.F., GOTTLIEB, A., NISSEN, N., STUTZMAN, L. & COOPER, M.R. (1982). Second malignant neoplasms in patients successfully treated for Hodgkin's disease: A cancer and leukaemia group B study. *Cancer Treat. Rep.*, **66**, 1035.

JONES, S.E., COLTMAN, C.A. & GROZEA, P.N. (1982). Conclusions from clinical trials of the Southwest Oncology Group. *Cancer Treat. Rep.*, **66**, 847.

KANT, J.A., HUBBARD, S.M., LONGO, D.L., SIMON, R.M., DEVITA, V.T. & JAFFE, E.S. (1986). The pathologic and clinical heterogeneity of lymphocyte depleted Hodgkin's disease. *J. Clin. Oncol.*, **4**, 284.

KAPLAN, E.L. & MEIER, P. (1978). Non-parametric estimation from incomplete observations. *J. Am. Statist. Assoc.*, **54**, 457.

KAY, R. (1983). Goodness of fit methods for the proportional hazards regression model: A review. *University of Sheffield Research Report*. 232/RK.

KLIMO, P. & CONNORS, J.M. (1985). MOPP/ABVD hybrid program: combination chemotherapy based on an early introduction of seven effective drugs for advanced Hodgkin's disease. *J. Clin. Oncol.*, **3**, 1174.

LONGO, D.L., YOUNG, R.C., WESLEY, M. & 4 others (1986). Twenty years of MOPP therapy for Hodgkin's disease. *J. Clin. Oncol.*, **4**, 1295.

NICHOLSON, W.M., BEARD, M.E.V., CROWTHER, D. & 5 others (1970). Combination chemotherapy in generalised Hodgkin's disease. *Br. Med. J.*, **3**, 7.

PETERSON, B.A., PAJAK, T.F., COOPER, M.R. & 5 others (1982). Effect of age on the therapeutic response and survival in advanced Hodgkin's disease. *Cancer Treat. Rep.*, **66**, 889.

PETO, R., PIKE, M.C., ARMITAGE, P. & 7 others (1977). Design and analysis of randomized clinical trials requiring prolonged observation of each patient. *Br. J. Cancer*, **35**, 1.

PILLAI, G.N., HAGEMEISTER, F.B., VELASQUEZ, W.S. & 4 others (1985). Prognostic factors for Stage IV Hodgkin's disease treated with MOPP with or without bleomycin. *Cancer*, **55**, 691.

RODGERS, R.W., FULLER, L.M., HAGEMEISTER, F.B. & 7 others. (1981). Reassessment of prognostic factors in Stage IIIb and IV Hodgkin's disease treated with MOPP and radiotherapy. *Cancer*, **42**, 2196.

SCHILLING, R.F., McKNIGHT, B. & CROWLEY, J.J. (1982). Prognostic value of serum lactate dehydrogenase level in Hodgkin's disease. *J. Lab. Clin. Med.*, **99**, 382.

SUTCLIFFE, S.B., WRIGLEY, P.F.M., PETO, J. & 5 others (1978). MVPP chemotherapy regimen for advanced Hodgkin's disease. *Br. Med. J.*, **1**, 679.

TESTER, W.J., KINSELLA, T.J., WALLER, B. & 4 others (1984). Second malignant neoplasms complicating Hodgkin's disease: The National Cancer Institute experience. *J. Clin. Oncol.*, **2**, 762.

WAGSTAFF, J., STEWARD, W.P., JONES, M. & 6 others (1986). Factors affecting remission and survival in patients with advanced Hodgkin's disease treated with MVPP. *Haemat. Oncol.*, **4**, 135.

YOUNG, C.W., STRAUSS, D.J., MYERS, J. & 8 others (1982). Multidisciplinary treatment of advanced Hodgkin's disease by an alternating chemotherapeutic regimen of MOPP/ABVD and low dose radiation restricted to originally bulky disease. *Cancer Treat Rep.*, **66**, 907.

YOUNG, R.C., CORDER, M.P., BERARD, C.W. & DEVITA, V.T. (1973). Immune alterations in Hodgkin's disease. *Arch. Int. Med.*, **131**, 446.